# Accuracy Evaluation of Indirect Bonding Techniques for Clear Aligner Attachments Using 3D-Printed Models: An In Silico and Physical Model-Based Study

**DOI:** 10.3390/ma18040780

**Published:** 2025-02-11

**Authors:** Cosmina Raluca Fratila, Luis Óscar Alonso-Ezpeleta, Manuel Poveda-Saenz, Giovanni Giovannini, Ana Belén Lobo-Galindo, Javier Flores-Fraile, Álvaro Zubizarreta-Macho

**Affiliations:** 1 Faculty of Dentistry, Alfonso X el Sabio University, 28691 Madrid, Spain; cfrat@myuax.com (C.R.F.); giovanni@uax.es (G.G.); amacho@uax.es (Á.Z.-M.); 2 Endodontics Section, Department of Stomatology, School of Dentistry, University of Zaragoza, 50009 Zaragoza, Spain; lalonezp@unizar.es; 3 Faculty of Medicine, CEU-San Pablo University, 28039 Madrid, Spain; manuelpoveda1@gmail.com; 4 Department of Surgery, Faculty of Medicine, University of Salamanca, 37008 Salamanca, Spain; alobogal@hotmail.com

**Keywords:** accuracy, attachments, orthodontics, clear aligners, digital, composite resin

## Abstract

An inaccurate bonding procedure of the attachments related to clear aligner systems could influence the predictability of tooth movement The aim of this study was to compare the positioning reliability of horizontal and vertical orthodontic clear aligner attachments. Materials and Methods: A total of 70 horizontal and 70 vertical orthodontic clear aligner attachments were bonded to five upper and five lower experimental anatomically based acrylic resin models with 14 clinical crowns each. The experimental anatomically based acrylic resin models were randomly distributed to the following study groups: Group A—horizontal orthodontic clear aligner attachments (*n* = 70) (HORIZONTAL) and Group B—vertical orthodontic clear aligner attachments (*n* = 70) (VERTICAL). Afterward, the orthodontic clear aligner attachments were digitally planned using orthodontic planning software, and orthodontic templates were manufactured by thermoforming on 3D-printed models in trilayer glycol-modified polyethylene terephthalate. Both horizontal and vertical orthodontic clear aligner attachments were put through an intra-oral scan to obtain a postoperative digital image, and PAPver, PCPver, PMVver, AUver, Alver, PPMhor, PPDhor, PMVhor, AMhor and ADhor cephalometric parameters were analyzed using a *t*-test or a non-parametric Mann–Whitney–Wilcoxon test. Results: The results showed that all cephalometric parameters showed statistically significant differences (*p* < 0.05) between the accuracy of the indirect bonding technique for horizontal and vertical orthodontic clear aligner attachments, except for the PAPver (*p* = 0.6079) and PMVhor (*p* = 0.5001) cephalometric parameters. Conclusions: The vertical orthodontic clear aligner attachments are more accurate than the horizontal orthodontic clear aligner attachments through the indirect bonding technique.

## 1. Introduction

Patient demand for aesthetic orthodontic treatments is continually increasing in adults and pediatric patients [1]; specifically, gender influences the perception of the aesthetics of the smile [2], with women being more critical than men and generating the most demands for orthodontic treatment. Moreover, Saccomanno et al. reported that 87.94% of patients are female and 72.57% of them are required to improve their aesthetics, while only 54.84% of male patients state this reason for consultation [3]. Therefore, dentists must have knowledge of the different types of aesthetic orthodontics to make a correct diagnosis and properly execute the treatment plan [4].

Furthermore, Baron reported on three types of orthodontic appliances with a high rate of aesthetic acceptance by patients: thermoplastic aligners, lingual orthodontics and aesthetic braces [5]; however, conventional multibracket appliance techniques do not have high acceptance by patients due to insufficient aesthetics, hygiene problems and quality of life (pain, discomfort when chewing, mouth injuries, etc.) [6,7].

A systematic review published in 2020 verified that fixed orthodontic treatment can have a negative impact on quality of life 24 h after placement of the appliance and up to three months after the start of treatment [8].

Thermoplastic aligners are more discreet and comfortable to wear and can be removed for eating and brushing teeth. Alami et al. evaluated in a descriptive cross-sectional study the perception and satisfaction of patients treated with transparent aligners, and the main reason for choosing this type of orthodontics was its “invisibility” (89.7%) [9]. The possibility of having correct oral hygiene at home is essential for periodontal patients, making this type of orthodontics their main choice if the duration of treatment is longer than 18 months [10]. Likewise, the risk of developing stain lesions, white spots or dental cavities is reduced; therefore, it is a recommended orthodontic treatment for patients with a high risk of cavities. Moreover, Abay et al. analyzed the prevalence of white spots during orthodontic treatment and observed that the number of patients who developed them after treatment with aligners (41.18%) was lower than who underwent fixed orthodontic treatment with self-ligating brackets (63.64%) and conventional brackets (52.94%) [11].

For all these reasons, interest in clear aligners has grown [7]. The treatment consists of wearing transparent thermoplastic splints individualized to each patient for a minimum of 20 h a day, except for eating and drinking [12]. In addition, computer-aided design is combined with three-dimensional virtual model correction software to organize the dental movements and correct malocclusion, obtaining a simulation of the results of the treatment before starting [13,14]. Currently, clear aligners can be used for a wide variety of complex malocclusions using various auxiliary resources such as attachments, mini-screws and elastics [7,15,16].

Moreover, attachments have undergone a great evolution over the years, since at the beginning they only had an ellipsoidal shape (horizontal for active intrusion movements and vertical for retention objectives). Later, rectangular attachments were introduced, which are distinguished by their shape: horizontal, vertical and beveled. In particular, beveled attachments have developed into beveled attachments tailored to the requirement for dental mobility. These attachments serve the same purpose as traditional ones, but they are positioned along the tooth’s vertical axis. Teardrop-shaped attachments are used to direct the canines’ derotation in situations of several rotations or when the correction is generally larger than two degrees. They are always customized based on the form, length and width of the teeth. Later additions include the “power ridge”, which is utilized to achieve proper vertical control of the incisor axis in numerous motions and enhance the correction of torque larger than three degrees. Similarly, to address deep bites, “bite ramps” were added as a horizontal accessory to the palatal surface of the upper front teeth; they were only inserted buccal in cross-bite situations.

In every situation, the accuracy with which the attachments are cemented and the composite material utilized have a significant impact on how well the aligner, attachment and tooth interact [17].

The nature of composite resin (flowable or conventional) influences the adhesion of attachments to the tooth and also to the shape accuracy. Likewise, the thermoplastic template used to reproduce the shape of the attachments is a fundamental factor for the precision and predictability of future tooth movement [18,19]. There are several risk factors for early failure of attachments and/or changes in their shape and, with it, a loss of retention and predictable movement of the aligners, both related to the operator and the patient. Regarding factors related to the operator, the choice of an appropriate composite with ideal properties represents a relevant aspect for the maintenance of the attachment [20]; hence, flowable composites or orthodontic bonding composites with the greatest amount of filler possible and a rigid transfer template for greater precision and strength of the composite bond are used [21]. In general, patient-related causes of attachment loss are wearing aligners for less than 18 h a day, the use of aligner seats or “chewies” and unilateral chewing. This represented 56.25% of the early loss of the attachment. However, tooth-related reasons resulting in attachment loss were 6.73% [22]. Likewise, it must be considered that the attachment can wear out. Li Q et al. demonstrated that the wear volume of the attachment gradually increases as the treatment time increases. The average wear volume was 0.813 mm^3^ after 8 months of treatment [23].

Currently, the scientific literature does not provide information related to the effect of the geometrical design of clear aligner attachments on the positioning reliability of clear aligner attachments, since an inaccurate positioning with respect to planning can lead to an unpredictable orthodontic treatment, undesirable complications and longer-lasting treatments. The present study provides practical implications with direct clinical application that could influence the predictability of tooth movement, making the clinical results of orthodontic treatment with clear aligners more precise.

The aim of this work was to compare the positioning reliability of horizontal and vertical orthodontic clear aligner attachments placed through indirect bonding technique, with a null hypothesis (H_0_) stating that no differences were shown between the positioning reliability of horizontal and vertical orthodontic clear aligner attachments placed through indirect bonding technique.

## 2. Materials and Methods

### 2.1. Study Design

A total of 70 horizontal and 70 vertical orthodontic clear aligner attachments (Spectra ST flow, Dentsply, Ballaigues, Switzerland) were bonded on 5 upper and 5 lower experimental anatomically based acrylic resin models (Ref. 20-8130-128, EpoxiCure^®^, Buehler, Lake Bluff, IL, USA) with 14 clinical crowns each, applying a photo-polymerized composite resin cement (Transbond™ XT, 3M ESPE™, Saint Paul, MN, USA) in the middle of the buccal surface of the crown, followed by 20 s of etching the buccal surface with 37% orthophosphoric acid (VOCOCID, VOCO GmbH, Cuxhaven, Germany) and 20 s of applying a photo-polymerized resin adhesive primer (Unitek Transbond™ XT, 3M ESPE™, Saint Paul, MN, USA) to replicate a clinical setting. The research was carried out at the Dental Centre of Innovation and Advanced Specialties at Alfonso X El Sabio University (Madrid, Spain), between September 2023 and March 2024. A randomized controlled study was conducted in accordance with the principles defined in the German Ethics Committee’s statement for the use of organic tissues in medical research (Zentrale Ethikkommission, 2003). Sample size determination was determined for a total of 140 samples (70 per group), which would provide 95% power in determining statistically significant differences in variables analyzed among the 2 groups (effect size: 0.70, alpha = 5%).

### 2.2. Experimental Procedure

**Figure 1:**
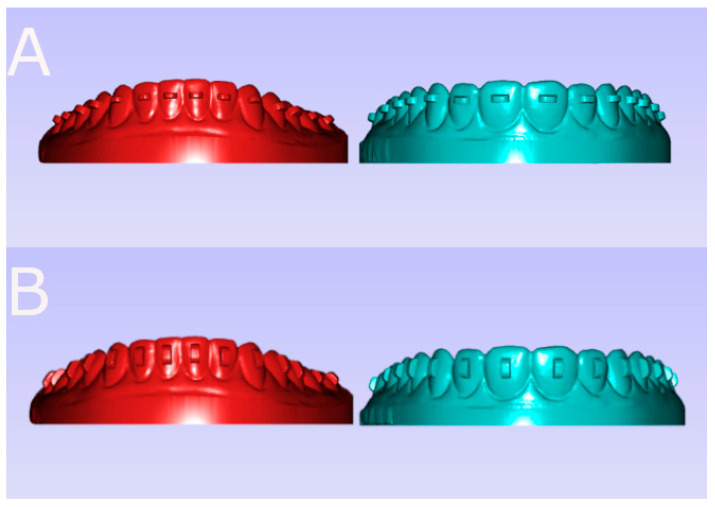
(**A**) Frontal view of the lower (red) and upper (green) experimental anatomically based acrylic resin models with the horizontal orthodontic clear aligner attachments planning and (**B**) frontal view of the lower (red) and upper (green) experimental anatomically based acrylic resin models with the vertical orthodontic clear aligner attachments planning.

The following research groups received the experimental anatomically based acrylic resin models at random (Epidat 4.1, Galicia, Spain): Group A, horizontal orthodontic clear aligner attachments (*n* = 70) (HORIZONTAL); and Group B, vertical orthodontic clear aligner attachments (*n* = 70) (VERTICAL). A 3D printing system (ProJet^®^ 6000. 3D Systems©, Rock Hill, SC, USA) was used to construct the upper and lower experimental anatomically based acrylic resin models, which were designed using 2D/3D computer-aided design/computer-aided engineering (CAD/CAE) (Midas FX+^®^, Milton Keynes, UK). Afterward, the orthodontic clear aligner attachment placement was digitally planned using orthodontic planning software (QuickSmile^®^, https://quicksmile.es/ (access on 27 November 2024), Madrid, Spain) (Figure 1).

Then, digital orthodontic templates were also designed using orthodontic planning software (QuickSmile^®^, Madrid, Spain). Subsequently, the orthodontic templates were manufactured by thermoforming on 3D-printed models (BioStar, Scheu Dental, Madrid, Spain) in 0.76 mm trilayer glycol modified polyethylene terephthalate (PET-G), following the manufacturer’s recommendations.

Subsequently, both horizontal and vertical orthodontic clear aligner attachments were performed by flowable composite resin (Spectra ST flow, Dentsply, Ballaigues, Switzerland) on both upper (from tooth 1.7 to 2.7) and lower (from tooth 3.7 to 4.7) acrylic resin models based on experimental anatomy placed in the middle of the clinical crown’s buccal surface without etching or resin glue application. In order to obtain an accurate standard tessellation language (STL) digital file, all experimental anatomically based acrylic resin models were then subjected to a postoperative digital impression using an intra-oral scan (iTero Element 5D Plus, Align Technology, Santa Clara, CA, USA) using 3D in-motion video imaging technology. In accordance with the manufacturer’s instructions, the occlusal plane was scanned first, and then the buccal and palatal surfaces, to capture the image.

### 2.3. Alignment Procedure

**Figure 2:**
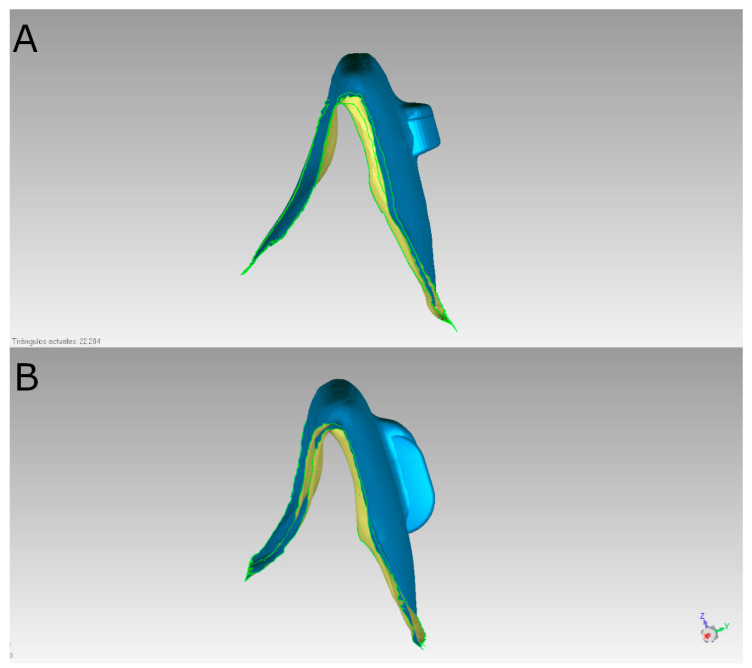
(**A**) Lateral views of the alignment procedure of the preoperative and postoperative STL digital files of horizontal and (**B**) vertical orthodontic clear aligner attachments in the buccal surface of tooth 1.1.

Subsequently, the STL digital files were imported into a reverse-engineering morpho-metric software program (3D Geomagic Capture Wrap, 3D Systems©, Rock Hill, SC, USA), where the teeth were individually segmented to facilitate the accurate alignment procedure between the STL digital files of the virtually planned positions of the digitalized horizontal and vertical orthodontic clear aligner attachments (QuickSmile^®^, Madrid, Spain) and the postoperative STL digital files of the same attachments bonded to the experimental anatomically based acrylic resin models, following the guidelines established by Brandão et al. [24]. The alignment procedure utilized the palatal surfaces of the anterior teeth and the occlusal and palatal surfaces of the posterior teeth, employing the best-fit algorithm (Figure 2).

### 2.4. Measurement Procedure

**Figure 3:**
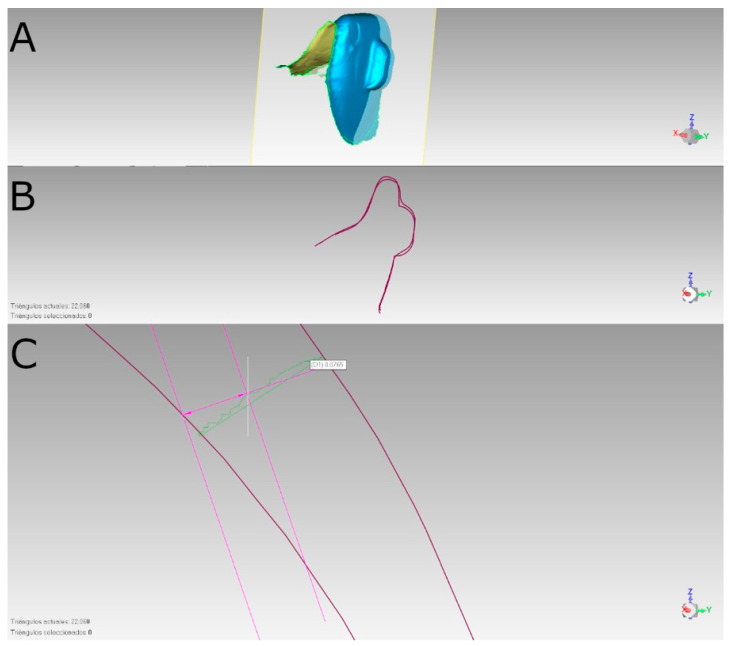
(**A**) Vertical plane crossing the aligned STL digital files of the vertical orthodontic clear aligner attachments in the buccal surface of tooth 1.1; (**B**) transversal plane of the aligned STL digital files of the vertical orthodontic clear aligner attachments in the buccal surface of tooth 1.1 and (**C**) Measurement procedure.

The following characteristics were measured on both the vertical and the horizontal cut planes following the alignment process (Figure 3).

The vertical cutting plane’s variables include the following:

PAPver: The greatest difference between the two apico-palatal attachment points (point–apico-palatal–vertical) of the physical and virtual models.

PCPver: point–coronal–palatal–vertical, the greatest difference between the two most coronal–palatal points of the attachments of the virtual and physical models.

PMVver: The greatest difference between the virtual and physical models’ attachments’ two middle vestibular points (point–middle vestibular–vertical)

AUver: The angle (angle–upper vertical) between the virtual and physical models’ upper horizontal and vertical attachment profiles.

ALver: The angle (angle–lower–vertical) between the virtual and physical models’ lower horizontal and vertical attachment profiles.

Horizontal cutting plane variables include:

PPMhor: The greatest difference between the virtual and physical models’ two most mesial–palatal attachment points (point–palatal–mesial–horizontal).

PPDhor: The greatest difference between the virtual and physical models’ two furthest disto–palatal attachment points (point–palatal–distal–horizontal).

PMVhor: Point–middle–vestibular–horizontal, the greatest difference between the two middle vestibular attachment points of the physical and virtual models.

AMhor: The angle–mesial–horizontal angle that forms between the virtual and physical models’ upper approximal attachment profiles.

ADhor: The angle (angle–distal–horizontal) established between the virtual and physical models’ approximate lower approximation attachment profiles.

### 2.5. Statistical Analysis

A table has been obtained with the summary statistics for each of the response variables based on the group: number of observations, mean, median, standard deviation and the minimum and maximum values. They have been represented graphically using box plots.

In addition, the means between groups were compared using a *t*-test (PCPver, PPMhor, PMVhor and ADhor) or a non-parametric Mann–Whitney–Wilcoxon test (PAPver, PMVver, AUver, ALver, PPDhor and AMhor) according to application criteria. Normality tests were carried out using the Shapiro–Wilk test. Statistical analysis was performed with the software: SAS v9.4, SAS Institute Inc., Cary, NC, USA. The statistical decisions have been made taking the value 0.05 as the level of significance.

## 3. Results

**Figure 4:**
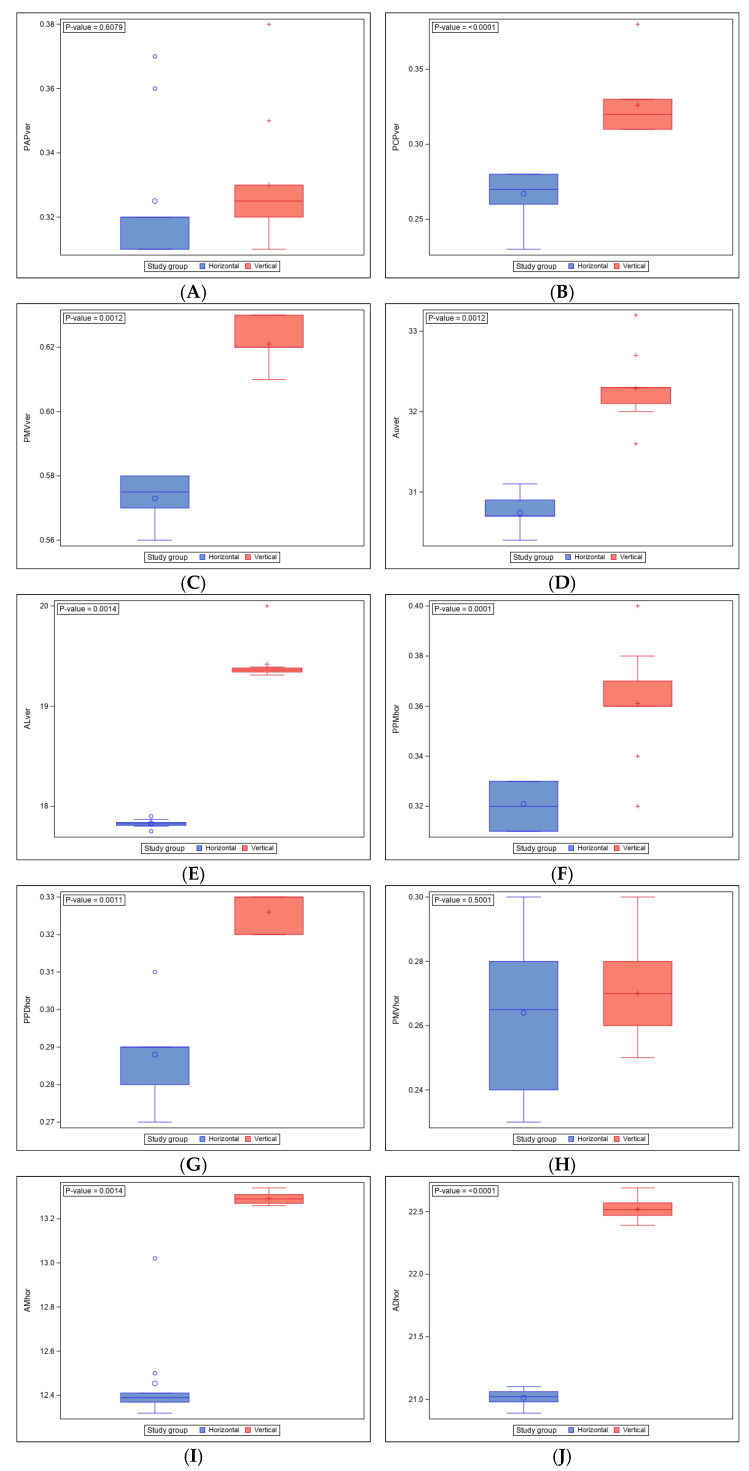
Box plot of the (**A**) PAPver linear measurement (mm), (**B**) PCPver linear measurement (mm), (**C**) PMVver linear measurement (mm), (**D**) AUver angle measurement (°), (**E**) ALver angle measurement (°), (**F**) PPMhor linear measurement (mm), (**G**) PPDhor linear measurement (mm), (**H**) PMVhor linear measurement (mm), (**I**) AMhor angle measurement (°) and (**J**) ADhor angle measurement (°) of the horizontal and vertical orthodontic clear aligner attachments. Horizontal line represents the median and “+,◦” represents the mean.

The mean, median and SD values for the PAPver linear measurement (mm) between the STL digital files of both horizontal and vertical orthodontic clear aligner attachments are displayed in Table 1 and Figure 4A.

A non-parametric Mann–Whitney–Wilcoxon test did not show statistically significant differences between the PAPver linear measurement (mm) of horizontal and vertical orthodontic clear aligner attachments (*p* = 0.6079) (Figure 4A).

The mean, median and SD values for the PCPver linear measurement (mm) between the STL digital files of both horizontal and vertical orthodontic clear aligner attachments are displayed in Table 1 and Figure 4B.

Student’s *t*-test showed statistically significant differences between the PCPver liner measurement (mm) of the horizontal and vertical orthodontic clear aligner attachments (*p* < 0.0001) (Figure 4B).

The mean, median and SD values for the PMVver linear measurement (mm) between the STL digital files of both the horizontal and vertical orthodontic clear aligner attachments are displayed in Table 1 and Figure 4C.

Th non-parametric Mann–Whitney–Wilcoxon test showed statistically significant differences between the PMVver liner measurement (mm) of horizontal and vertical orthodontic clear aligner attachments (*p* = 0.0012) (Figure 4C).

The mean, median and SD values for the AUver angle measurement (°) between the STL digital files of both the horizontal and vertical orthodontic clear aligner attachments are displayed in Table 1 and Figure 4D.

The non-parametric Mann–Whitney–Wilcoxon test showed statistically significant differences between the AUver angle measurement (mm) of the horizontal and vertical orthodontic clear aligner attachments (*p* = 0.0012) (Figure 4D).

The mean, median and SD values for the ALver angle measurement (°) between the STL digital files of both the horizontal and vertical orthodontic clear aligner attachments are displayed in Table 1 and Figure 4E.

The non-parametric Mann–Whitney–Wilcoxon test showed statistically significant differences between the ALver angle measurement (mm) of horizontal and vertical orthodontic clear aligner attachments (*p* = 0.0014) (Figure 4E).

The mean, median and SD values for the PPMhor linear measurement (mm) between the STL digital files of both the horizontal and vertical orthodontic clear aligner attachments are displayed in Table 1 and Figure 4F.

Student’s *t*-test showed statistically significant differences between the PPMhor linear measurement (mm) of the horizontal and vertical orthodontic clear aligner attachments (*p* = 0.0001) (Figure 4F).

The mean, median and SD values for the PPDhor linear measurement (mm) between the STL digital files of both the horizontal and vertical orthodontic clear aligner attachments are displayed in Table 1 and Figure 4G.

The non-parametric Mann–Whitney–Wilcoxon test showed statistically significant differences between the PPDhor linear measurement (mm) of the horizontal and vertical orthodontic clear aligner attachments (*p* = 0.0011) (Figure 4G).

The mean, median and SD values for the PMVhor linear measurement (mm) between the STL digital files of both the horizontal and vertical orthodontic clear aligner attachments are displayed in Table 1 and Figure 4H.

Student’s *t*-test did not show statistically significant differences between the PMVhor linear measurement (mm) of the horizontal and vertical orthodontic clear aligner attachments (*p* = 0.5001) (Figure 4H).

The mean, median and SD values for the AMhor angle measurement (°) between the STL digital files of both the horizontal and vertical orthodontic clear aligner attachments are displayed in Table 1 and Figure 4I.

The non-parametric Mann–Whitney–Wilcoxon test showed statistically significant differences between the AMhor angle measurement (mm) of the horizontal and vertical orthodontic clear aligner attachments (*p* = 0.0014) (Figure 4I).

The mean, median and SD values for the ADhor angle measurement (°) between the STL digital files of both the horizontal and vertical orthodontic clear aligner attachments are displayed in Table 1 and Figure 4J.

Student’s *t*-test showed statistically significant differences between the ADhor angle measurement (mm) of the horizontal and vertical orthodontic clear aligner attachments (*p* < 0.0001) (Figure 4J).

The results of this study indicate that the most of the variables of the descriptive statistics between STL digital files of both the horizontal and vertical orthodontic clear aligner attachments show significant differences between virtual and in vivo precision of attachments reproduction; however, PAPver and PMVhor linear measurements (mm) of the horizontal and vertical orthodontic clear aligner attachments did not show significant differences. These differences may be due to slight changes experienced in the attachments after removal of the attachment cementing splint; that is, greater resistance and greater traction may be experienced when removing a retainer with horizontal attachments than one with vertical attachments, due to the smaller base size.

## 4. Discussion

The results obtained in the present study refuted the null hypothesis (H_0_), which stated that no differences would be shown between the positioning reliability of horizontal and vertical orthodontic clear aligner attachments placed through indirect bonding.

The accuracy of the attachments is one of the key components of clear aligner therapy to obtain more precision on tooth movement [25].

Clear aligners have revolutionized orthodontic therapy, offering an esthetic treatment in addition to correct dental malocclusion in patients of all ages [26,27]. Technological developments in aligner materials and techniques, especially 3D technology for planning dental movements, allow for the tooth movements of the teeth along the three planes of space more predictably. The type of movements with clear aligners are the following: (a) traslational movements: molar distalization, displacement of teeth to extraction sites and vestíbulo-lingual/mesio-distal tooth displacement; (b) extrusion movement; (c) intrusion movement; (d) tipping movement: uncontrolled tipping and controlled tipping; torque and rotation movement [28]. Clear aligners are driven by two systems to obtain tooth movements. The first is a displacement-driven system which favors less complicated movements, like tipping and control of minor rotations, because the termoplastic aligner designed by software is placed at the final location of the tooth, allowing the tooth to move or displace until it aligns with the aligner [29]. Buccolingual inclination of upper and lower incisors and produce satisfied clinical results [30]. The second is a force-driven system which operates on biomechanical principles. The amount and type of force applied depend on the shape of the aligners, with each tooth receiving a specific magnitude and type of force as determinate by software [29]. Clear aligners were not effective enough to produce adequate occlusal contacts, controlling tooth torque and retention [30].

Castroflorio et al. [18]. report that tipping and torque movements are the most difficult to control with aligners, and the loss of information increases moving toward the distal portion of the aligner. For most tooth angulation and inclination (mesio-distal tipping and bucco-lingual inclination), the worst conditions are revealed in the first and second molars on both arches.

Treatment duration, accuracy, attachability, cost, accessibility and biocompatibility are some of the issues that clear aligners must deal with [25,26,27,28,29]. The length of treatment depends on the degree of malocclusion and patient compliance [26]. Different levels of therapy accuracy have been found in clinical investigations, indicating the need for more investigation. In aligner treatment results, prediction models have demonstrated an accuracy of around 78% [25,30,31]. Two crucial elements need to be taken into account in order to enhance aligner retention and system performance: the use of power ridges and attachments, as well as the careful selection of aligner material [32,33]. In contrast to the virtual setup, attachments are essential to clear aligner procedures because they provide torque, rotate teeth and affect treatment results [31,34,35]. Shape attachments determine how an aligner tooth moves, and any disparities might result in a crown-pushing force [36]. The mechanical characteristics of the aligner surface are improved by improving composite attachments, which increases aligner efficacy [37]. Issues such as heat deformation, material expansion and shrinkage might result in thickness variations that impact the aligner’s final result and prevent the proper occlusion [38,39].

Aligners now have better mechanical qualities, biocompatibility and overall quality thanks to ongoing advancements in material development. Because of the release of monomers during thermoforming, certain thermoplastic polymers used in aligners may have cytotoxic consequences. ISO 20795-2 [26,40] specifies the mechanical and physical characteristics of dental resins used in orthodontic devices in order to guarantee their quality and safety. As a result, aligners are now less intrusive and more affordable and produce results that are on par with those of other available treatments [25].

Definitely, clear aligners represent a paradigm shift in modern orthodontics, providing a more convenient solution for malocclusion than conventional fixed orthodontic treatment because they facilitate good oral hygiene maintenance, are esthetic and comfortable and grant the patient satisfaction of occlusion and functionality [28]; however, Meto et al. reported microbial plaque formation on orthodontic clear aligners and recommended a copper–calcium hydroxide-based compound to remove microbial plaque formation [41].

This study used horizontal and vertical shape attachment, flowable composite and PET-G transfer templates, which are considered gold standards in the study of attachment reproduction [16], and the sample size is greater than that of the few other studies in the literature evaluated. However, the present investigation has limitations related to the in vitro experimental design of the study.

Previous studies have been conducted to assess the influence of different variables on the accuracy of orthodontic clear aligner attachment placement. Topsakal et al. analyzed the accuracy of the ovoid, hemi-ellipsoid and vertical rectangular attachments produced using digital light-processing three-dimensional printing technologies with 25 µm, 75 µm and 125 µm layer thickness and concluded that the layer thickness of the 3D printer is a crucial factor in determining attachment accuracy [25]; moreover, Fiorillo et al. retrospectively evaluated the accuracy of dental rotational movements using clear aligners with different attachment configurations and stated that attachment configurations to clear aligners improve rotational accuracy, but not significantly; specially optimized attachments showed the highest median accuracy (70%), followed by rectangular (65%) [42]; in addition, Brandão et al. evaluated the 3D accuracy of attachment positioning using in-house templates made with either polyethylene terephthalate glycol (PETG) or ethylene-vinyl acetate (EVA) and either pressure or vacuum thermoforming machines and stablished that the inaccuracy of the attachment positioning was slight and the vacuum and pressure thermoplastification machines showed no difference in attachment positioning accuracy [24]; furthermore, Valeri et al. assessed the accuracy of the process of attachment bonding in aligner treatments using two types of transfer templates and two light-curing resin-based composites (Transbond™ XT Light Cure Paste Adhesive) usually used in orthodontics and stated that a resin-based composite with a rigid transfer template is always associated with significant accuracy and minor dispersion [43], although Chen et al. concluded that the operation time of Z350XT Flowable and SonicFill was shorter than Z350XT composite resin [44], and D’Antò et al. stated that flowable composite resin cements were appropriate for attachment fabrication; moreover, the fidelity of attachment reproduction was similar among them, and the orthodontic composite showed more overflow when compared with the flowable one [45]. Nevertheless, the present scientific literature fails to directly compare the impact of the geometric design of the clear aligner attachments on the precision and dependability of their positioning.

The present study provides practical implications with direct clinical application that could influence the predictability of tooth movement, making the clinical results of orthodontic treatment with clear aligners more precise. However, the study also showed limitations, since the researchers considered only one density of composite resin cement and a clear aligner system in the study to standardize the sample; moreover, the present laboratory-based study used 3-D printed models with different physical properties than natural teeth models. Furthermore, the authors advocate the creation of randomized controlled clinical trials incorporating different densities of composite resin cements and a clear aligner system for further research.

## 5. Conclusions

The vertical orthodontic clear aligner attachments are more accurate than the horizontal orthodontic clear aligner attachments through the indirect bonding technique; therefore, the authors recommend selecting attachments whose positioning is more reliable with respect to planning; especially in cases requiring mesiodistal inclination, mesiodistal translation or intrusion tooth movements for lower incisors, leading to a more predictable orthodontic treatment, avoiding complications and longer lasting treatments.

## Figures and Tables

**Table 1:** Descriptive statistics of PAPver linear measurement (mm), PCPver linear measurement (mm), PMVver linear measurement (mm), AUver angle measurement (°), ALver angle measurement (°), PPMhor linear measurement (mm), PPDhor linear measurement (mm), PMVhor linear measurement (mm), AMhor angle measurement (°) and ADhor angle measurement (°) between the STL digital files of both the horizontal and vertical orthodontic clear aligner attachments.

PAPver Linear Measurement (mm)						
Study Group	*n*	Mean	Median	SD	Minimum	Maximum
Horizontal	70	0.330	0.325	0.021	0.310	0.380
Vertical	70	0.325	0.320	0.022	0.310	0.370
PCPver linear measurement (mm)						
Horizontal	70	0.326	0.320	0.021	0.310	0.380
Vertical	70	0.267	0.270	0.016	0.230	0.280
PMVver linear measurement (mm)						
Horizontal	70	0.621	0.620	0.007	0.610	0.630
Vertical	70	0.573	0.575	0.008	0.560	0.580
AUver angle measurement (°)						
Horizontal	70	32.290	32.300	0.425	31.600	33.200
Vertical	70	30.740	30.700	0.190	30.400	31.100
ALver angle measurement (°)						
Horizontal	70	19.418	19.360	0.206	19.310	20.000
Vertical	70	17.827	17.825	0.040	17.750	17.900
PPMhor linear measurement (mm)						
Horizontal	70	0.361	0.360	0.021	0.320	0.400
Vertical	70	0.321	0.320	0.009	0.310	0.330
PPDhor linear measurement (mm)						
Horizontal	70	0.326	0.330	0.005	0.320	0.330
Vertical	70	0.288	0.290	0.010	0.270	0.310
PMVhor linear measurement (mm)						
Horizontal	70	0.270	0.270	0.016	0.250	0.300
Vertical	70	0.264	0.265	0.023	0.230	0.300
AMhor angle measurement (°)						
Horizontal	70	13.292	13.290	0.025	13.260	13.340
Vertical	70	12.454	12.390	0.204	12.320	13.020
ADhor angle measurement (°)						
Horizontal	70	22.520	22.515	0.084	22.390	22.690
Vertical	70	21.013	21.020	0.062	20.890	21.100

SD: standard deviation.

## Data Availability

The original contributions presented in this study are included in the article. Further inquiries can be directed to the corresponding author.

## References

[1] Paglia L., Marzo G. (2023). Aligners, can my child use them too?. Eur. J. Paediatr. Dent..

[2] Bolas-Colvee B., Tarazona B., Paredes-Gallardo V., Arias-De Luxan S. (2018). Relationship between perception of smile esthetics and orthodontic treatment in Spanish patients. PLoS ONE.

[3] Saccomanno S., Saran S., Laganà D., Mastrapasqua R.F., Grippaudo C. (2022). Motivation, Perception, and Behavior of the Adult Orthodontic Patient: A Survey Analysis. BioMed Res. Int..

[4] Alhasyimi A.A., Ayub A., Farmasyanti C.A. (2024). Effectiveness of the Attachment Design and Thickness of Clear Aligners during Orthodontic Anterior Retraction: Finite Element Analysis. Eur. J. Dent..

[5] Baron P. (2014). Les appareils orthodontiques invisibles et presque invisibles [Invisible and almost invisible orthodontic appliances]. L’Orthodontie Française.

[6] Li Q., Du Y., Yang K. (2023). Comparison of pain intensity and impacts on oral health-related quality of life between orthodontic patients treated with clear aligners and fixed appliances: A systematic review and meta-analysis. BMC Oral Health.

[7] Mendes Ribeiro S.M., Aragón M.L.S.C., Espinosa D.D.S.G., Shibasaki W.M.M., Normando D. (2024). Orthodontic aligners: Between passion and science. Dent. Press J. Orthod..

[8] Zhang B., Huang X., Huo S., Zhang C., Zhao S., Cen X., Zhao Z. (2020). Effect of clear aligners on oral health-related quality of life: A systematic review. Orthod. Craniofacial Res..

[9] Alami S., Sahim S., Hilal F., Essamlali A., El Quars F. (2022). Perception and satisfaction of patients treated with orthodontic clear aligners. Open Access Lib J..

[10] Chhibber A., Agarwal S., Yadav S., Kuo C.-L., Upadhyay M. (2018). Which orthodontic appliance is best for oral hygiene? A randomized clinical trial. Am. J. Orthod. Dentofac. Orthop..

[11] Abay F., Buyuk S.K., Korkmaz Y.N. (2022). Prevalence of white spot lesions during clear aligner therapy: A systematic review. Australas. Orthod. J..

[12] Galluccio G. (2021). Is the use of clear aligners a real critical change in oral health prevention and treatment?. Clin. Ter..

[13] Zheng M., Liu R., Ni Z., Yu Z. (2017). Efficiency, effectiveness and treatment stability of clear aligners: A systematic review and meta-analysis. Orthod. Craniofacial Res..

[14] AlMogbel A. (2023). Clear Aligner Therapy: Up to date review article. J. Orthod. Sci..

[15] Ma X.Q., Xiang F., Fan M.Y., Song Y., Wang X.H., Zhang L., Qian W.H. (2022). Clinical efficacy of the combination of miniscrew with clear aligner in controlling the roller coaster effect. Shanghai Kou Qiang Yi Xue.

[16] Bellocchio A.M., Portelli M., Ciraolo L., Ciancio E., Militi A., Peditto M., Barbera S., Nucera R. (2023). Evaluation of the Clinical Variables Affecting Attachment Reproduction Accuracy during Clear Aligner Therapy. Materials.

[17] Putrino A., Barbato E., Galluccio G. (2021). Clear Aligners: Between Evolution and Efficiency—A Scoping Review. Int. J. Environ. Res. Public Health.

[18] Castroflorio T., Sedran A., Parrini S., Garino F., Reverdito M., Capuozzo R., Mutinelli S., Grybauskas S., Vaitiekūnas M., Deregibus A. (2023). Predictability of orthodontic tooth movement with aligners: Effect of treatment design. Prog. Orthod..

[19] Nucera R., Dolci C., Bellocchio A.M., Costa S., Barbera S., Rustico L., Farronato M., Militi A., Portelli M. (2022). Effects of Composite Attachments on Orthodontic Clear Aligners Therapy: A Systematic Review. Materials.

[20] Gazzani F., Bellisario D., Quadrini F., Danesi C., Alberti A., Cozza P., Pavoni C. (2022). Light-curing process for clear aligners’ attachment reproduction: Comparison between two nanocomposites cured by the auxiliary of a new tool. BMC Oral Health.

[21] Jedliński M., Mazur M., Greco M., Belfus J., Grocholewicz K., Janiszewska-Olszowska J. (2023). Attachments for the Orthodontic Aligner Treatment-State of the Art-A Comprehensive Systematic Review. Int. J. Environ. Res. Public Health.

[22] Yaosen C., Mohamed A.M., Jinbo W., Ziwei Z., Al-Balaa M., Yan Y. (2021). Risk Factors of Composite Attachment Loss in Orthodontic Patients During Orthodontic Clear Aligner Therapy: A Prospective Study. BioMed Res. Int..

[23] Li Q., Yang K. (2024). Surface wear of attachments in patients during clear aligner therapy: A prospective clinical study. Prog. Orthod..

[24] Brandão N.M.C.B., Maia R.M., Gomes V.M., Resende C., Antunes A.N.D.G., Souki B.Q. (2024). Bonding positional accuracy of attachments and marginal adaptation of in-house aligners—A quality improvement laboratory study. Orthod. Craniofacial Res..

[25] Topsakal K.G., Gökmen Ş., Yurdakurban E., Duran G.S., Görgülü S. (2023). The effect of layer thıckness on the accuracy of the dıfferent ın-house clear alıgner attachments. Clin. Oral Investig..

[26] Narongdej P., Hassanpour M., Alterman N., Rawlins-Buchanan F., Barjasteh E. (2024). Advancements in Clear Aligner Fabrication: A Comprehensive Review of Direct-3D Printing Technologies. Polymers.

[27] Aljabaa A.H. (2020). Clear Aligner Therapy—Narrative Review. J. Int. Oral Health.

[28] Moutawakil A. (2021). Biomechanics of aligners: Literature Review. Adv. Dent. Oral Health.

[29] Katib H.S., Hakami A.M., Albalawei M., Alhajri S.A., Alruwaily M.S., Almusallam M.I., Alqahtani G.H. (2024). Stability and Success of Clear Aligners in Orthodontics: A Narrative Review. Cureus.

[30] Yu X., Li G., Zheng Y., Gao J., Fu Y., Wang Q., Huang L., Pan X., Ding J. (2022). ‘Invisible’ orthodontics by polymeric ‘clear’ aligners molded on 3D-printed personalized dental models. Regen. Biomater..

[31] Izhar A., Singh G., Goyal V., Singh R., Gupta N., Pahuja P. (2019). Comparative Assessment of Clinical and Predicted Treatment Outcomes of Clear Aligner Treatment: An in Vivo Study. Turk. J. Orthod..

[32] Upadhyay M., Arqub S.A. (2022). Biomechanics of Clear Aligners: Hidden Truths & First Principles. J. World Fed. Orthod..

[33] Lombardo L., Palone M., Carlucci A., Siciliani G. (2020). Clear Aligner Hybrid Approach: A Case Report. J. World Fed. Orthod..

[34] Weir T. (2017). Clear Aligners in Orthodontic Treatment. Aust. Dent. J..

[35] Elshazly T.M., Keilig L., Salvatori D., Chavanne P., Aldesoki M., Bourauel C. (2022). Effect of Trimming Line Design and Edge Extension of Orthodontic Aligners on Force Transmission: An in Vitro Study. J. Dent..

[36] Ammann R., Tanner C., Schulz G., Osmani B., Nalabothu P., Töpper T., Müller B. (2022). Three-Dimensional Analysis of Aligner Gaps and Thickness Distributions, Using Hard X-ray Tomography with Micrometer Resolution. J. Med. Imaging.

[37] Ho C.-T., Huang Y.-T., Chao C.-W., Huang T.-H., Kao C.-T. (2021). Effects of Different Aligner Materials and Attachments on Orthodontic Behavior. J. Dent. Sci..

[38] Hartshorne J., Wertheimer M.B. (2022). Emerging Insights and New Developments in Clear Aligner Therapy: A Review of the Literature. AJO-DO Clin. Companion.

[39] Panayi N.C. (2023). Directly Printed Aligner: Aligning with the Future. Turk. J. Orthod..

[40] (2013). Dentistry—Base polymers. Part 2: Orthodontic Base Polymers.

[41] Meto A., Colombari B., Castagnoli A., Sarti M., Denti L., Blasi E. (2019). Efficacy of a Copper-Calcium-Hydroxide Solution in Reducing Microbial Plaque on Orthodontic Clear Aligners: A Case Report. Eur. J. Dent..

[42] Fiorillo G., Campobasso A., Croce S., Hussain U., Battista G., Lo Muzio E., Mandelli G., Ambrosi A., Gastaldi G. (2024). Accuracy of clear aligners in the orthodontic rotational movement using different attachment configurations. Orthod. Craniofacial Res..

[43] Valeri C., Aloisio A., Mummolo S., Quinzi V. (2022). Performance of Rigid and Soft Transfer Templates Using Viscous and Fluid Resin-Based Composites in the Attachment Bonding Process of Clear Aligners. Int. J. Dent..

[44] Chen W., Qian L., Qian Y., Zhang Z., Wen X. (2021). Comparative study of three composite materials in bonding attachments for clear aligners. Orthod. Craniofacial Res..

[45] D’Antò V., Muraglie S., Castellano B., Candida E., Sfondrini M.F., Scribante A., Grippaudo C. (2019). Influence of Dental Composite Viscosity in Attachment Reproduction: An Experimental In Vitro Study. Materials.

